# 

**Published:** 1914-08-08

**Authors:** 


### CONTENTS.

| | PAGE |
|---|---|
| **War and Insanity** ... ... ... ... ... | 509 |
| **The Administrative Problem of Workhouse Nursing** ... ... ... ... ... ... | 510 |
| **Hospital and Institutional News** ... ... ... | 511 |
| Hospitals and the War—The British Red Cross Society's Preparations—Surgeons' Services in Request—Attendants "an Utterly Unskilled Class"—The Supply of Drugs by Poor-Law Doctors—Increased Salaries under the Asylums Board—The Assistant Medical Officers' Views—The "Tone" of the Association—The Tuberculosis Scheme for London—A Patient's Ingenuity—An Employers' Plan for Raising Subscriptions—Secretarial Changes at Lloyd Hospital, Bridlington—The West End Hospital and Rebuilding—Cottage Hospital Pay-wards—The Dewsbury Infirmary Endowment Fund—Suggested Extensions at Swansea Hospital—The Rally of the Hospital Saturday Fund—Dr. Arthur Newsholme and Sanatoria—The Extension of Altrincham Hospital—Hospital Schools—Institutional Co-operation—An Improvement at Hanwell Mental Hospital—The Doctor in National Education—Bath Guardians Urged to Rebuild—Sanatorium Patients and Sick Pay—Important Bequests to Brighton Hospitals—The Crisis and the Drug Market. | |
| **The Electrical and Radio-Therapeutic Department of the Cancer Hospital (Free), London** | 517 |
| II.—Details of Equipment and Cost. | |
| **New Infirmary Scheme at Bristol** ... ... | 520 |
| The Reorganisation of the Indoor Medical Work. | |
| **Obituary** ... ... ... ... ... ... ... ... | 520 |
| **Medical Services and Hospitals of the Crown** | 521 |
| A Survey of Our Organisation. | |
| **New Developments at Leicester** ... ... ... | 522 |
| **National Insurance Act Developments** ... ... | 523 |
| The Second Annual Report. | |
| **Americans on the Voluntary System** ... ... | 525 |
| The Criticisms of Visiting Surgeons. | |
| **Round the World** ... ... ... ... ... ... | 526 |
| Fellow-Workers and their Doings. | |
| **Metropolitan Hospital Sunday Fund** ... ... | 527 |
| The Distribution Committee. | |
| Report of the Committee of Distribution to the Council. | |
| **Medical Appointments** ... ... ... ... | 530 |
| **The Department of Modern Nursing** ... ... | 531 |
| North London or University College Hospital. | |
| **The Editor's Letter-Box** ... .. ... ... | 532 |
| "Reply to the Criticisms of a Patient"—The Secretaryship of St. Mark's. | |
| **The Institutional Library** ... ... ... ... | 533 |
| A Mental Hospital Handbook—Institutional Segregation—A Pocket Guide to Nursing. | |
| **The Profession of Medicine** ... ... ... | 534 |
| The Clinical Congress. | |
| **The Causation of Apoplexy** ... ... ... | 536 |
| **The Institutional Worker** ... ... (Suppl.) | 1 |
| Dinner Tins. | |
| Points in Institutional Housekeeping. | |
| Questions for August and September. | |
| Questions Invited. | |
| **Manager's Notices** ... ... ... ... (Cover) | 2 |

